# Clinical significance of contrast extravasation on computed tomography immediately after thermal ablation for hepatic tumors

**DOI:** 10.1186/s12876-025-04025-4

**Published:** 2025-07-01

**Authors:** Dong Kyu Kim, Joon Ho Kwon, Kichang Han, Juil Park, Gyoung Min Kim, Man-Deuk Kim, Jong Yun Won

**Affiliations:** 1 Department of Radiology, Yongin Severance Hospital, Research Institute of Radiological Science, Yonsei University College of Medicine, Yongin, Korea; 2 Department of Radiology, Severance Hospital, Research Institute of Radiological Science, Yonsei University College of Medicine, 50-1 Yonsei-ro Seodaemun-gu, Seoul, 03722 Korea

**Keywords:** Hepatic tumor, Ablation, Contrast extravasation, Computed tomography, Angiography

## Abstract

**Background:**

To evaluate the clinical significance of contrast extravasation observed on post-ablation computed tomography (CT) performed immediately following thermal ablation of hepatic tumors.

**Methods:**

Between October 2014 and December 2023, 1,274 patients with 1,745 primary or metastatic hepatic tumors underwent ablation, including radiofrequency ablation, microwave ablation, and cryoablation. Among them, 30 patients (median age: 66 years) with contrast extravasation observed on post-ablation CT scans were retrospectively analyzed. The pre- and post-ablation hemoglobin and hematocrit levels were measured. Local tumor progression-free survival (LTPFS) and overall survival (OS) rates were evaluated.

**Results:**

Among the 30 patients, angiography was performed in 6 patients. Contrast extravasation was observed on angiography in only two patients; contrast extravasation from the right inferior phrenic artery and intercostal artery was noted, and successful transarterial embolization was achieved. Conservative management was considered adequate without additional treatment in 28 of 30 patients. No significant differences were observed between the 1 day before and after ablation hemoglobin (12.9 g/dL; 12.0–13.8 g/dL vs. 12.5 g/dL; 11.5–13.8 g/dL, *P* = 0.102) and hematocrit (38.3%; 36.0–40.1% vs. 37.0%; 34.8–39.2%, *P* = 0.100) levels. During a mean follow up period of 23.3 ± 17.8 months, the LTPFS rates were 96.4% and 84.3% at 1 and 2 years, respectively. The OS rate after the procedure was 96.7%.

**Conclusion:**

The presence of contrast extravasation on post-ablation CT was not clinically significant, when extravasation confined to intrahepatic or venous origins. However, transarterial embolization is required if contrast extravasation is detected in the extrahepatic arteries.

## Background

Percutaneous thermal ablation treatments, including radiofrequency ablation (RFA), microwave ablation (MWA), and cryoablation, are widely used and accepted as effective and safe treatment modalities for unresectable primary and metastatic hepatic tumors [1–3]. Despite the favorable aspects of ablation treatment, such as low morbidity and mortality rates, it has also been associated with various adverse events. Among the adverse events related to ablation procedures, bleeding complications, including localized or subcapsular hematoma, hemoperitoneum, and hemothorax, can be serious and life-threatening [4–6].

Performing post-ablation computed tomography (CT) immediately after ablation can help identify local residual tumors and avoid delays in the diagnosis and management of bleeding [7]. Previous research has demonstrated the safety and efficacy of transarterial angiography and embolization in controlling bleeding complications associated with hepatic ablation without requiring surgery [8–10].

However, little published information is available on the clinical significance of post-ablation contrast extravasation and the need of transarterial angiography for bleeding management following hepatic ablation, as most of the previous studies included case reports and focused on bleeding following RFA [9, 11]. Therefore, this study aimed to evaluate the clinical significance of contrast extravasation observed on post-ablation CT images taken immediately after ablation treatments, including RFA, MWA, and cryoablation, for primary and secondary hepatic tumors.

## Methods

### Study patients

This single-center retrospective study was approved by the Institutional Review Board, and the requirement for informed consent was waived.

**Fig. 1:**
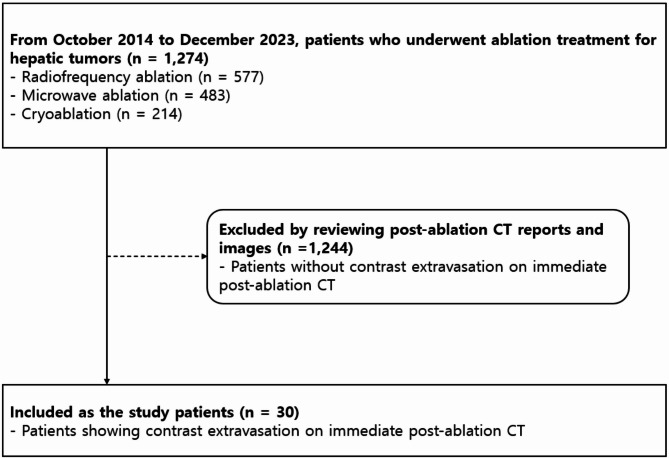
Flow chart of study population. CT, computed tomography

Between October 2014 and December 2023, 1,274 patients with 1,745 hepatic tumors underwent ablations, including RFA, MWA, and cryoablation. We identified cases of contrast extravasation by reviewing all immediate post-ablation CT reports and images over the study period. Among these patients, 30 patients (median age, 66 years; range, 45–83 years) who showed contrast extravasation on post-ablation CT performed immediately after the procedure were included in this study (Fig. 1). Closed hemodynamic monitoring was performed for at least 30 min after the ablation. Among the 30 study patients, those with suspected hemodynamic instability, such as systolic arterial pressure < 90mmHg or acute decrease in blood pressure with compensatory increase in heart rate determined by physicians’ clinical assessment, underwent transarterial angiography. The electronic medical charts of each patient were reviewed by a single radiologist to obtain the clinical data and laboratory findings.

Of the study patients, 16 (53.3%), 12 (40.0%), and 2 (6.7%) patients were treated with MWA, RFA, and cryoablation, respectively. The tumors were hepatocellular carcinomas (HCC), metastases from colorectal cancers, and metastasis from pancreatic neuroendocrine tumors in 26 (86.7%), 3 (10.0%), and 1 (3.3%) patient(s), respectively. HCC was diagnosed based on the typical imaging findings on liver dynamic CT or magnetic resonance imaging (MRI), such as arterial hyperenhancement and portal venous or delayed phase washout, with elevated levels of serum α-fetoprotein and/or vitamin protein induced by vitamin K absence-II [12]. There were 32 hepatic tumors in 30 patients, and the median number of tumors was one (range,1–2). Median tumor size was 1.5 cm (range:0.9–3.0 cm). The locations of the hepatic tumors were as follows: segment II, *n* = 1; segment III, *n* = 1; segment IV, *n* = 2; segment V, *n* = 6; segment VI, *n* = 6; segment VII, *n* = 10; and segment VIII, *n* = 6.

### Ablation procedure

Ablation procedures for hepatic tumors were performed by interventional radiologists in a hybrid angiography suite equipped with an angio-CT system that incorporated a multidetector CT scanner (INFX-8000 C combined with an Aquilion 128 channel CT scanner, Toshiba Medical Systems Corp, Japan) and an angiography system. Thirty minutes prior to the procedure, all the patients received 25 mg of pethidine hydrochloride (Pethidine, Myungmoon Pharm, Korea) intramuscularly and local anesthesia was administered using 10–20 mL of 1% lidocaine (Lidocaine, Daihan Pharm, Korea). While most ablation procedures were performed under ultrasound (US)-guidance, in cases where the tumor is difficult to visualize on US, the procedure is performed under CT guidance. During the procedure, vital signs were monitored using pulse oximetry and electrocardiography.

The choice of ablation modality among RFA, MWA, and cryoablation was left to the discretion of the referring physicians and interventional radiologists, who were fully informed about the advantages and disadvantages of each modality at a multidisciplinary conference at our institution. RFA was performed using a 17-gauge cooled-tip needle (Cool-tip RF ablation system, Valleylab, Boulder. Co, USA). After an initial power application of 50 W, the power was increased in 10 W increments per minute. MWA was performed using a 13-gauge antenna (Emprint, Medtronic, MN, USA) at a power of 70–100 W. During RFA and MWA, the operators determined the number of needles, power of the generator, and ablation time, depending on the shape and size of the lesion. Track ablation was routinely performed during needle removal. Cryoablation was performed using a cryoprobe (IceRod i-Thaw 1.5, [17 G] straight cryoablation probe, Gail Medical, Israel). The number of cryoprobes was determined by the operators according to the shape and size of the lesion, and at least two cryoprobes were used. The ablation process consisted of a double-thaw cycle with a 10-minute freezing period and 8-minute thawing period in each cycle.

The endpoint of ablation was determined by immediate post-procedural imaging; after completion of the ablation, three-phase dynamic contrast-enhanced CT was performed to evaluate whether the ablation zone sufficiently covered the tumor and to assess any immediate postprocedural complications. If the ablation margin was considered adequate on CT, the procedure was finalized. Contrast pooling on arterial-phase of post-ablation CT at the ablation site was indicative of active bleeding. Ablation procedures and evaluation of post-ablation CT including confirming contrast extravasation were performed by experienced interventional radiologists over 10-year experience.

### Transarterial angiography and embolization procedure

In the patients who underwent transarterial angiography, vascular access was achieved through the right common femoral artery under US guidance. Common hepatic artery and superior mesenteric artery angiography were performed using a 5-Fr RH catheter (Cook, Bloomington, IN, USA) and a 0.035-inch hydrophilic guidewire (Terumo, Japan). Furthermore, selective angiography was performed to determine whether the vessels along the electrode insertion route were injured. Superselection of the bleeding vessels was performed using a 2.2-Fr microcatheter (Progreat, Terumo) and a 0.014-inch microguidewire (Meister, Asahi, Japan). Contrast extravasation or the presence of a pseudoaneurysm was considered indicative of active bleeding. If active bleeding was identified on angiography, transarterial embolization was performed using NBCA (N-butyl 2-cyanoacrylate) (B. Braun, Melsungen, Germany) as the embolic material mixed with Lipiodol (Guerbet, Roissy, France). Transarterial angiography and embolization procedures were also performed by experienced interventional radiologists over 10-year experience.

### Definitions and data analysis

Coagulopathy was defined as an international normalized ratio [INR] of prothrombin time [PT] ≥ 1.5 or platelet counts ≤ 50,000/µL. Nodules at high-risk locations were identified by measuring the distance from the edge of the tumor to the hepatic dome, extrahepatic organs, and hepatic capsule. Nodules adjacent to extrahepatic organs were defined as those located within 10 mm of the lungs, heart, gallbladder, kidneys, gastrointestinal tract, or diaphragm [13]. Subcapsular lesions were defined as those located ≤ 10 mm from the liver surface [14].

Local tumor progression (LTP) was defined as the appearance of new tumor foci at the ablative margins. The degree of tumor necrosis was assessed based on the modified response evaluation criteria for solid tumor [15]. Adverse events were classified as mild, moderate, severe, life-threatening or disabling, and fatal, according to the Society of Interventional Radiology Adverse Events Classification system [16].

### Statistical analysis

All the statistical analyses were performed using the SPSS software (version 25.0; IBM, Armonk, New York, USA) for Windows. Continuous variables are expressed as median and range. Pre-ablation laboratory findings were obtained one day before the procedure and post-ablation laboratory findings were obtained one day after the procedure; pre- and post-ablation laboratory levels were compared using the Wilcoxon signed-rank test. The LTP-free survival (LTPFS) and overall survival (OS) were calculated using the Kaplan–Meier method.

## Results

### Study patients

The baseline characteristics of the study participants are summarized in Table 1. There was only one patient with coagulopathy (platelet count = 48000/uL, PT INR = 1.13). More than half of the hepatic tumors (21 of 32, 65.6%) were at high-risk locations. Of the 30 patients showing contrast extravasation on post-ablation CT, 2 patients showed extrahepatic arterial extravasation and the remaining cases showed small-volume extravasation along the needle tract or within the subcapsular space. Among them, 6 patients underwent transarterial angiography due to suspicious hemodynamic instability; of the 6 patients, 3 patients had undergone RFA and 3 patients MWA, and 4 of 6 nodules were at high-risk locations. The remaining 24 of 30 patients who did not undergo transarterial angiography were hemodynamically stable.

**Table 1:** Patients’ baseline characteristics

Characteristic	Value
No. of patients	30
Age (years), median, range	66, 45–83
Sex	
Men	25 (83.3)
Women	5 (16.7)
Coagulopathy	1 (3.3)
Liver cirrhosis	17 (56.7)
Child-Pugh score	
A	24 (80.0)
B	6 (20.0)
Treatment modality	
RFA	12 (40.0)
MWA	16 (53.3)
Cryoablation	2 (6.7)
Management of contrast extravasation on CT	
Angiography	6 (20.0)
Conservative management without angiography	24 (80.0)
Duration of Hospitalization (days), median, range	4, 3–10
No. of hepatic tumors	
Total	32
Median, range	1, 1–2
Pathology of hepatic tumors	
HCC	27 (84.4)
Metastasis from colorectal cancer	4 (12.5)
Metastasis from pancreas NET	1 (3.1)
Maximal diameter of tumor (cm), median, range	1.5, 0.9–3.0
Tumors at high-risk location†	21 (65.6)

Values in parentheses are percentages

RFA, radiofrequency ablation; MWA, microwave ablation; CT, computed tomography; HCC, hepatocellular carcinoma; NET, neuroendocrine tumor

† Tumors at high-risk location: nodules located within 10 mm from the lung, heart, gallbladder, kidney, gastrointestinal tract, diaphragm, or liver capsule

### Transarterial angiography and clinical outcomes

**Fig. 2:**
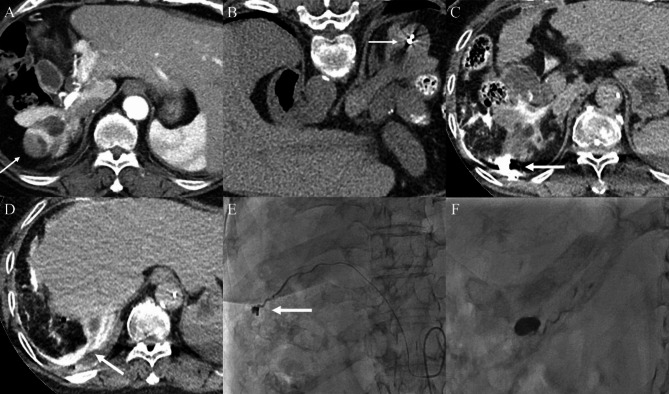
A case of a 66-year-old female having bleeding after microwave ablation (MWA). (**A**) Pre-procedure liver dynamic computed tomography (CT) revealed hepatocellular carcinoma (HCC) in the segment VII subcapsular area (arrow). (**B**) After microwave antenna placement (arrow), non-contrast CT was performed to determine if the lesion was appropriately targeted. (**C, D**) After completion of ablation, post-ablation three-phase dynamic contrast-enhanced CT was performed, and contrast extravasation was observed (arrows). (**E, F**) On transarterial angiography, active bleeding from the right inferior phrenic artery was noted (arrow); thus, embolization with a mixture of NBCA and Lipiodol was performed

**Fig. 3:**
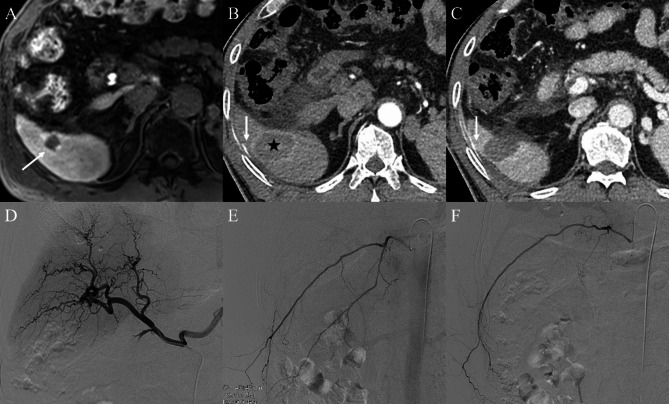
A case of a 66-year-old male showing contrast extravasation on post-ablation computed tomography (CT) following radiofrequency ablation (RFA). (**A**) Pre-procedure liver dynamic magnetic resonance imaging (MRI) revealed hepatocellular carcinoma (HCC) in the segment VI subcapsular area (arrow). (**B, C**) After completion of ablation, post-ablation three-phase dynamic contrast-enhanced CT was performed. The tumor was completely included in the ablation zone (asterisk); however, contrast extravasation was observed (arrows). (**D, E, F**) Nevertheless, no evidence of active bleeding was observed on transarterial angiography, including hepatic and intercostal arteriograms

All six patients showed contrast extravasation on post-ablation CT before undergoing transarterial angiography. However, on angiography, active bleeding was observed in only two of the six patients; contrast extravasation from the right inferior phrenic artery and right intercostal artery was noted, and successful transarterial embolization was achieved in these two cases (Fig. 2). However, in 4 of the 6 patients, no evidence of bleeding on angiography was noted (Fig. 3). The details of the procedures and outcomes are summarized in Table 2.

**Table 2:** Details of transarterial angiography and the clinical outcomes

Case	Sex/ Age	Hepatic tumor	Tumor Location	Ablation treatment	Transarterial Angiography	Adverse event after angiography	Duration of Hospitalization	Local tumor recurrence	Death	Follow up period (months)
1	M/83	HCC	Segment IV Dome*	RFA	No bleeding	No	4 days	No	No	35.6
2	M/62	HCC	Segment VIII Dome*	RFA	No bleeding	No	3 days	No	No	14.7
3	M/51	HCC	Segment V No high risk	MWA	Embolization for right ICA	No	3 days	No	No	15.8
4	M/40	Rectal cancer	Segment VII No high risk	MWA	No bleeding	No	6 days	No	No	24.4
5	M/66	HCC	Segment VI Subcapsular*	RFA	No bleeding	No	10 days	No	No	6.7
6	F/66	HCC	Segment VII Subcapsular*	MWA	Embolization for right IPA	No	4 days	No	No	10.4

M, male; F, female; HCC, hepatocellular carcinoma; RFA, radiofrequency ablation; MWA, microwave ablation; ICA, intercostal artery; IPA, inferior phrenic artery

* “Dome” and “Subcapsular” are specific types of high-risk locations, whereas “No high risk” indicates the tumor was > 10 mm away from the capsule or critical structures

Except for 2 patients treated with transarterial embolization, the remaining 28 of 30 patients were followed up with conservative management such as bed rests, vital sign monitoring, and follow-up laboratory tests for 1–2 days, without additional treatment. There were no significant differences between the hemoglobin (12.9 g/dL; 12.0–13.8 g/dL vs. 12.5 g/dL; 11.5–13.8 g/dL, *P* = 0.102) and hematocrit (38.3%; 36.0–40.1% vs. 37.0%; 34.8–39.2%, *P* = 0.100) levels 1 day before and after ablation in 28 patients. There were no adverse events related to angiography or embolization. Notably, no patient in this series required blood transfusion, and there were no re-bleeding events within 1 month. The median hospitalization duration was 4 days (range, 3–10 days), and all the patients were alive and discharged after confirming their hemodynamic stability. There was no any delay nor additional procedure that could have impacted tumor control in patients who experienced bleeding. Furthermore, the patients who underwent transarterial angiography with/without embolization did not exhibit longer recovery time and did not undergo any additional imaging studies beyond routine follow-up examinations.

**Fig. 4:**
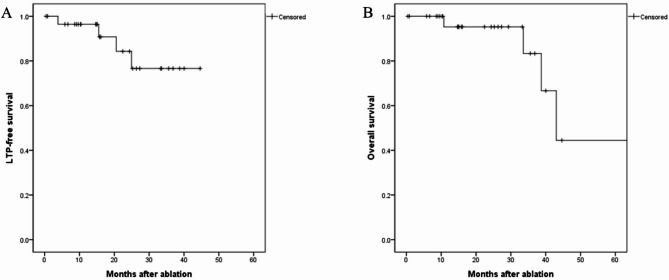
Kaplan–Meier curves of (**A**) local tumor progression-free survival (LTPFS) and (**B**) overall survival in 30 patients who underwent hepatic ablation treatments. The LTPFS rates were 96.4% and 84.3% at 1 and 2 years, respectively. The overall survival rates were 96.7% at 1 and 2 years

During a mean follow up period of 23.3 ± 17.8 months, LTP occurred in 4 patients (13.3%). In the four patients with LTP, additional RFA was performed in one patient, and transarterial chemoembolization (TACE) was performed in three patients. The LTPFS rates were 96.4% at one year and 84.3% at two years following ablation treatment. Moreover, four patients (13.3%) died during the follow-up period; one patient died 10 months after ablation, and three patients died at least 3 years after ablation. The OS rate was 96.7% 1 and 2 years after the procedure (Fig. 4). In addition, no LTPs or deaths were observed in the six patients who underwent transarterial angiography. As a reference, the 1-year and 2-year LTPFS rates in the control group without contrast extravasation were 95.8% and 82.7%, respectively. The 1-year and 2-year OS rates in this group were 95.0% and 90.4%, respectively.

## Discussion

Most of previous research on hepatic ablation have primarily focused on treatment efficacy, complications, and factors associated with these outcomes. However, little published information is available on the clinical significance of post-ablation contrast extravasation and the necessity of transarterial angiography for bleeding management following hepatic ablation. The present study evaluated the clinical significance of contrast extravasation observed on post-ablation CT images, immediately after ablation treatment for hepatic tumors. Of the 30 study patients who showed contrast extravasation on post-ablation CT, six patients with suspicious hemodynamic instability, such as decreased blood pressure or increased heart rate, underwent transarterial angiography. However, on angiography, active bleeding was seen in only two of the six patients from the right inferior phrenic artery and intercostal artery. Except for two patients treated with transarterial embolization, the remaining 28 of 30 patients were followed up with conservative management, which was considered sufficient without additional treatment. Furthermore, the LTPFS rates of the study patients were 96.4% and 84.3% at 1 and 2 years after ablation treatment; thus, post-ablation contrast extravasation did not diminish the treatment efficacy for hepatic tumors.

The incidence of contrast extravasation after ablation treatment for hepatic tumors was 1.7% (30 of 1745 tumors) in this study, which was comparable to the reported rate of bleeding after hepatic ablation ranging from 0.32 to 1.6% [17–19]. Nodules proximal to the hepatic dome, extrahepatic organs, and hepatic capsule are considered high-risk locations that could increase the incidence of complications following ablation. Moreover, liver cirrhosis can also increase the risk of bleeding, given that patients with cirrhosis are often predisposed to developing thrombocytopenia [10, 19]. In this study, more than half of the study patients had liver cirrhosis (17/30, 56.7%) and tumors at high-risk location (21/32, 65.6%).

In the present study, conservative management was sufficient for most patients, even when contrast extravasation was observed on post-ablation CT. Prior studies documented cases of puncture-related complications, such as mild bleeding and/or subcapsular hematoma caused by puncture into the hepatic artery and though the liver capsule during transjugular intrahepatic portosystemic shunt or transjugular liver biopsy, which require multiple needle passages, that were managed by conservative treatment with/without transfusion [20–22]; thus, the results of our study could be interpreted within a similar framework. Moreover, one potential explanation for the absence of active bleeding on transarterial angiography, despite its presence on CT, could be attributed to venous bleeding. Contrast extravasation on post-ablation CT can occur owing to arterial or venous injuries during the procedure. If contrast extravasation occurs due to arterial bleeding, it can also be detected using transarterial angiography. Similarly, venous angiogram may be required if extravasation occurs owing to venous bleeding [23]. However, venous bleeding is usually self-limiting and is treated conservatively with or without blood transfusions [24]; thus, an additional venous angiogram was not performed in this study.

However, in addition to the injuries to the hepatic vessels or capsules when performing hepatic ablation treatment, one more thing to consider is the possibility of puncture-related injury to extrahepatic arteries, such as the intercostal or inferior phrenic artery. Little information is available concerning bleeding from the extrahepatic artery after hepatic ablation; however, it could be fatal. Therefore, as in the cases in this study, transarterial angiography and embolization are required as first-line treatment options if patients show contrast extravasation from the extrahepatic artery [25]. However, in this study, only two patients showed extrahepatic artery bleeding. Therefore, further studies with larger sample sizes are warranted.

The LTPFS and OS rates were 96.4% and 96.7%, and 84.3% and 96.7% at 1 and 2 years, respectively. These outcomes are comparable to those observed in the control group without post-ablation contrast extravasation, which demonstrated LTPFS rates of 95.8% and 82.7%, and OS rates of 95.0% and 90.4% at 1 and 2 years, respectively. Furthermore, the results of our study are also comparable to those of previous research on ablation treatment reporting LTP rates ranging from 9.7 to 24.0% at 1 year and 3.1–33.3% at 2 years following the procedures [26–29]. Therefore, post-ablation contrast extravasation is not only unrelated to the treatment efficacy for hepatic tumors but also has no association with patient OS after ablation. In addition, no LTP or deaths were observed in the six patients who underwent transarterial angiography.

Additionally, performing multiphase contrast-enhanced CT immediately after ablation enables assessment of the ablation zone and any complications that may arise following the procedure [30]. If the ablation zone does not adequately cover the tumor, additional ablation treatment can be administered. Similarly, emergency angiography can be performed promptly if there are signs of bleeding. In our study, because of the immediate post-ablation CT scans, massive arterial bleeding was prevented by performing transarterial angiography and embolization of the extrahepatic artery.

Our study had several limitations. One of the main limitations of this study is the lack of a matched control group without contrast extravasation. Although LTPFS and OS rates appeared comparable between patients with and without post-ablation contrast extravasation, the absence of direct comparison limits interpretation of the clinical relevance. The retrospective and selective case design introduces potential selection bias, and comparisons based on external literature rather than within the study itself reduce the clarity of the findings. Therefore, as our study was not designed to analyze risk factors for bleeding after hepatic ablation, we acknowledge that it cannot determine predictors of hemorrhage. Furthermore, due to the lack of a control group, we recognize that it is not possible to draw definitive conclusions regarding whether bleeding had any impact on treatment outcomes; further prospective studies with appropriate control groups are necessary to confirm these observations. Second, the number of patients with post-ablation contrast extravasation was small, comprising only 1.7% of all ablation cases during the study period. While this reflects the rarity of significant post-procedural bleeding, it also limits the statistical power and generalizability of our results. Furthermore, the incidence of 1.7% reflects immediate post-ablation contrast extravasation, and although delayed bleeding events are rare, none were observed in this series. Moreover, the single-center design and use of a hybrid angio-CT suite for immediate post-ablation imaging may also reduce the generalizability of the findings. Multi-center studies with larger sample sizes are needed to validate the findings of this study. Third, the amount and extent of contrast extravasation were not quantitatively assessed in this study. Contrast extravasation was categorized in a binary manner (present or absent), whereas in clinical practice, the extent and severity of bleeding may be important factors in decision-making. Future studies could benefit from quantifying CT findings such as hematoma volume or the rate of contrast leakage, to better understand their relationship with the need for interventions. Fourth, the follow-up period was relatively short; thus, long-term outcomes should be further investigated. However, the short-term outcomes were consistent with those of previous reports. Finally, this study presented the results of ablation treatment, including RFA, MWA, and cryoablation, instead of evaluating the outcomes separately. Further studies with larger sample sizes for each ablation treatment are warranted.

## Conclusions

In conclusion, the presence of contrast extravasation on post-ablation CT is not clinically significant as most patients only required conservative treatment, when extravasation is confined to intrahepatic or venous origins. However, transarterial embolization is required if contrast extravasation is detected in the extrahepatic artery.

## Data Availability

The datasets used and/or analysed during the current study are available from the corresponding author on reasonable request.
